# Strength-balance supplemented with computerized cognitive training to improve dual task gait and divided attention in older adults: a multicenter randomized-controlled trial

**DOI:** 10.1186/1471-2318-14-134

**Published:** 2014-12-15

**Authors:** Eva van het Reve, Eling D de Bruin

**Affiliations:** Department of Health Sciences and Technology, Institute of Human Movement Sciences and Sport, ETH Zürich, Wolfgang-Pauli-Str. 27, 8093 Zürich, Switzerland; Department of Epidemiology, CAPHRI School for Public Health and Primary Care, PO Box 616, 6200 MD Maastricht, Netherlands; Department of Epidemiology, Centre for Evidence Based Physiotherapy, PO Box 616, 6200 MD Maastricht, Netherlands

**Keywords:** Cognitive-motor training, Dual task costs, Divided attention, Cognitive functions, Executive functions, Exercise, Fall prevention

## Abstract

**Background:**

Exercise interventions often do not combine physical and cognitive training. However, this combination is assumed to be more beneficial in improving walking and cognitive functioning compared to isolated cognitive or physical training.

**Methods:**

A multicenter parallel randomized controlled trial was conducted to compare a motor to a cognitive-motor exercise program. A total of 182 eligible residents of homes-for-the-aged (n = 159) or elderly living in the vicinity of the homes (n = 23) were randomly assigned to either strength-balance (*SB)* or strength-balance-cognitive (*SBC)* training. Both groups conducted similar strength-balance training during 12 weeks. *SBC* additionally absolved computerized cognitive training. Outcomes were dual task costs of walking, physical performance, simple reaction time, executive functions, divided attention, fear of falling and fall rate. Participants were analysed with an intention to treat approach.

**Results:**

The 182 participants (mean age ± SD: 81.5 ± 7.3 years) were allocated to either *SB* (n = 98) or *SBC* (n = 84). The attrition rate was 14.3%. Interaction effects were observed for dual task costs of step length (preferred walking speed: F(1,174) = 4.94, *p* = 0.028, η2 = 0.027, fast walking speed: F(1,166) = 6.14, *p* = 0.009, η2 = 0.040) and dual task costs of the standard deviation of step length (F(1,166) = 6.14, *p* = 0.014, η2 = 0.036), in favor of *SBC*. Significant interactions in favor of *SBC* revealed for in gait initiation (F(1,166) = 9.16, *p* = 0.003, η2 = 0.052), ‘reaction time’ (F(1,180) = 5.243, p = 0.023, η^2^ = 0.028) & ‘missed answers’ (F(1,180) = 11.839, *p* = 0.001, η^2^ = 0.062) as part of the test for divided attention. Within-group comparison revealed significant improvements in dual task costs of walking (*preferred speed*; velocity (*p* = 0.002), step time (*p* = 0.018), step length (*p* = 0.028), *fast speed*; velocity (*p* < 0.001), step time (*p* = 0.035), step length (*p* = 0.001)), simple reaction time (*p* < 0.001), executive functioning (Trail making test B; *p* < 0.001), divided attention (*p* < 0.001), fear of falling (*p* < 0.001), and fall rate (*p <* 0.001).

**Conclusions:**

Combining strength-balance training with specific cognitive training has a positive additional effect on dual task costs of walking, gait initiation, and divided attention. The findings further confirm previous research showing that strength-balance training improves executive functions and reduces falls.

**Trial registration:**

This trial has been registered under ISRCTN75134517

**Electronic supplementary material:**

The online version of this article (doi:10.1186/1471-2318-14-134) contains supplementary material, which is available to authorized users.

## Background

The progressive and dynamic aging process is characterized by functional and cognitive changes that often lead to physical performance deficits and deteriorations in walking. These changes occur even in the absence of overt diseases. Potential consequences are increased risk for falls, loss of independence in activities of daily living, and poor quality of life [1–5]. Functional dependence in older adults is associated with increased health care costs and mortality [6–8]. Minimizing falls is a common concern of many interventions as a third of people aged 65 and older and half of those aged 85 and older sustain falls each year, from which 10% result in serious consequences [9, 10]. One key factor in staying independent and maintaining mobility is, therefore, to enhance walking ability in older adults.

The general health protecting influence of physical activity in relation to muscular, skeletal, metabolic and cardiovascular functions is well documented [11–17]. The effect of physical [18] and cognitive [19–22] activity on brain functioning has also been recognized. Physical activity, for example, has been suggested reducing the incidence of dementia or cognitive deterioration [23–25], and is related to enhancements in cognitive functioning and brain plasticity [26–30]. Cognitive interventions resulted in improved cognitive speed [31], attention [32], and concentration [31]. Thus, cognitive functions are amenable both through physical and cognitive exercise, even in old age [23, 33–37].

Disparate lines of research converge on the notion that sensorimotor and cognitive aging are linked to each other in old age [38], and that daily tasks such as walking are dependent on both sensorimotor processes and higher level cognitive functions [39]. In the past walking has primarily been seen as representing an automated and reflex-controlled process [40, 41], which remains automatic when not deviating from learned programs [42]. However, older adults with cognitive impairments are exposed to falls, even when their motor functions are fairly intact [43, 44]. Recent literature suggests that the impact of sensorimotor impairments on falls is in part moderated by executive functions (EF) [45]. A review on this topic summarizes the interplay between EF, attention and gait [46]. Among healthy older adults, victims of falls performed poorly on EF and attention-demanding tasks [40, 47, 48], and the ability to pay attention seems to be an important requirement for walking that also influences the risk for falling [49]. Individuals with poor EF in turn have reduced gait speed [50], are more prone to falls [51] and have an increased risk of mortality [52]. EF has also been shown to associate with higher gait variability, which marks unsteadiness and inconsistency in walking, and likewise increases fall risk [53–56]. For minimized stride-to-stride fluctuation in gait an intact neural control system appears to be required [53]. A further walking aspect that is associated with higher level sensorimotor functions is gait initiation, and difficulties to initiate gait are related to disorders in the frontal lobe [1].

Divided attention, one component of executive functions, and some aspects of selective attention seem to be especially impaired in the aging process [57]. Dual-task related gait changes result from the competition interference between two attention-demanding tasks [58], and studies of cognitive changes during the aging process indicate that older adults’ ability to divide attention is decreased [59]. Compared to other specific components of executive functions, divided attention especially associates with spatial and temporal dual task cost characteristics of gait [60].

Basic components of a motor intervention program aiming to improve gait function in older adults are strength and balance exercises [61–64]. Training attention and executive function also improves gait [65, 66]. However, two recent reviews that focused on the interplay between physical functions and cognition concluded that it seems important to combine motor and cognitive therapy into clinical practice to enable older adults to move safer in their physical environment [46, 67] and that computerized interventions seem promising for this purpose [67]. Such an approach was tested in a pilot study, where traditional strength-balance training got complemented with computerized cognitive training of attention [68]. Cognitive-motor training tended to improve gait and foot reaction time to a greater extent than motor training alone. Because of the small sample size the association remained undetermined. There is a need for more studies on this topic with larger sample sizes [36, 46], and also for studies that address the effects of preventive interventions on cognitive performance [36] and, thereby, link the cognitive component to falls [46]. This study, therefore, aimed to further explore the additional effect of the supplemented cognitive training in a sufficiently powered trial. This randomized controlled trial was designed to examine the effects of exercise training and combined exercise and cognitive training on the physical and cognitive functioning of older adults. We hypothesized that both training groups would show significant improvements on measures of physical and cognitive functioning and, that the combined training group (exercise and cognitive training) would show greater walking function and cognitive improvements than the exercise-only training group.

## Methods

### Trial design

This study was a multicenter parallel randomized controlled clinical trial (trial registration: ISRCTN75134517). The study was carried out from March 2011 to December 2013. Participants were recruited from 14 homes-for-the-aged in Switzerland (n = 13) and Germany (n = 1). Permission of the ethical committees of the Cantons Berne, Zurich, Lucerne, St Gall, Argovia in Switzerland and Rhineland-Palatinate in Germany was received prior to study commencement. All participants provided written informed consent prior to participating in the study. The CONSORT Statement is used for reporting [69].

### Participants

Eligible residents of the homes-for-the-aged and interested autonomous living adults living in the vicinity of the homes were invited to attend an information session where the content of the intervention program and study design were explained. Based on the pilot study [68], where a 46% recruitment rate was reported, we estimated 467 potential participants from the 14 homes-for-the-aged. A sample of 192 residents of the homes and 23 autonomous living adults living in the vicinity of the homes indicated interest to participate. Participants were included when older than 65 years, scoring a minimum of 22 points on the Mini-Mental State Examination (MMSE), able to walk 20 meters with or without aids, free of rapidly progressive illness, acute illness or unstable chronic illness. Thirty-three subjects had to be excluded (MMSE n = 9, health problems n = 16, motivation problems n = 8). Hence, 182 individuals fulfilled all criteria. They were randomly allocated to either the strength-balance *SB* group (n = 94) or the strength-balance-cognitive *SBC* group (n = 88) using simple (unrestricted) randomisation [70] based on a random-number table. Four participants that were not able to conduct the cognitive training due to vision problems were manually allocated to the *SB* group after randomization. Thus, we reported 98 participants in *SB* and 84 participants in *SBC* after this adaptation. Individuals who met the initial eligibility criteria took part in a personal questionnaire based interview to screen for cognitive and health problems. Subjects who stopped doing their exercises any time during the 12 weeks of the program were defined as drop-outs.

### Sample size calculation

The sample size calculation for the number of participants is based on the primary outcome measure in the pilot study for the DTC of step duration and DTC of step length [68]. In order to avoid a type I or II error an estimated sample size of 64 (DTC of step duration) respectively 45 (DTC of step length) participants per group for a two group pre test – post test design was required, resulting in 80% power at an α-level of 0.05. To account for attrition over time, the required sample size was increased by 15% to 74 respectively 52 participants per group.

### Motor intervention program

**Figure 1:**
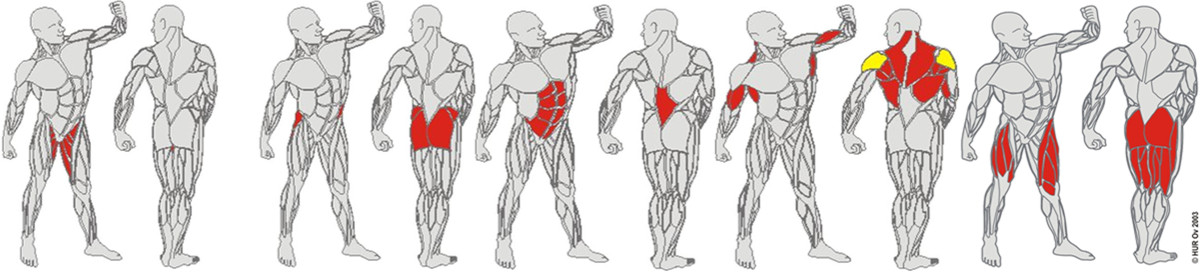
**The trained muscle groups of the strength training.** (Ab HUR Oy, 67100 Kokkola, Finland, http://www.hur.fi).

All participants performed an exercise program consisting of twice-weekly thirty minutes progressive resistance training on age-adapted machines and 10 minutes balance training during twelve weeks. Characteristics of age-adapted machines include a stepless increase or decrease of the resistance, restriction of range of motion through range limiters, ergonomic seats and, through this, a reduction of stress on vulnerable joints. Almost all of the homes trained with our preferred equipment using air-pressure as resistance (Ab HUR Oy, 67100 Kokkola, Finland (http://www.hur.fi). The requirement of the machines of the few homes that used weight stack machines was that they allowed increase or decrease of resistance in small steps of around 2-5 kg, depending on muscle group trained. The intervention was provided face to face to 4 to 6 participants at a time. The mix of strength training and balance exercises focusing on lower extremity muscle function was chosen to optimize transfer to functional tasks of daily living [71, 72]. Intensity, progression and duration of the program were based on previously published recommendations [11, 63, 73, 74]. Perceived exertion was obtained using the Borg’s scale of perceived exertion [75], and progression based on the participant’s statements. The muscle groups of the hip extensors, ab- and adductors, knee flexors and extensors, ankle dorsi- and plantar-flexors, abdominal- and back muscles as well as rhomboid muscles were trained (Figure 1). Additionally, one legged stance training, tandem standing and walking, walking on heels, backward and sideward walking, turns, sit-to stand-transfers and knee squats were executed. The balance program was performed using air-filled balance cushions (diameter 34 cm) (Sissel Schweiz, 8904 Aesch, http://www.sissel.ch), and consisted of static and dynamic functional exercises (e.g. standing on one leg, walking over cushions) [76]. Flexibility exercises followed each training session to maintain or improve the range of motion that is necessary for activities of daily living.

### Cognitive intervention program

**Figure 2:**
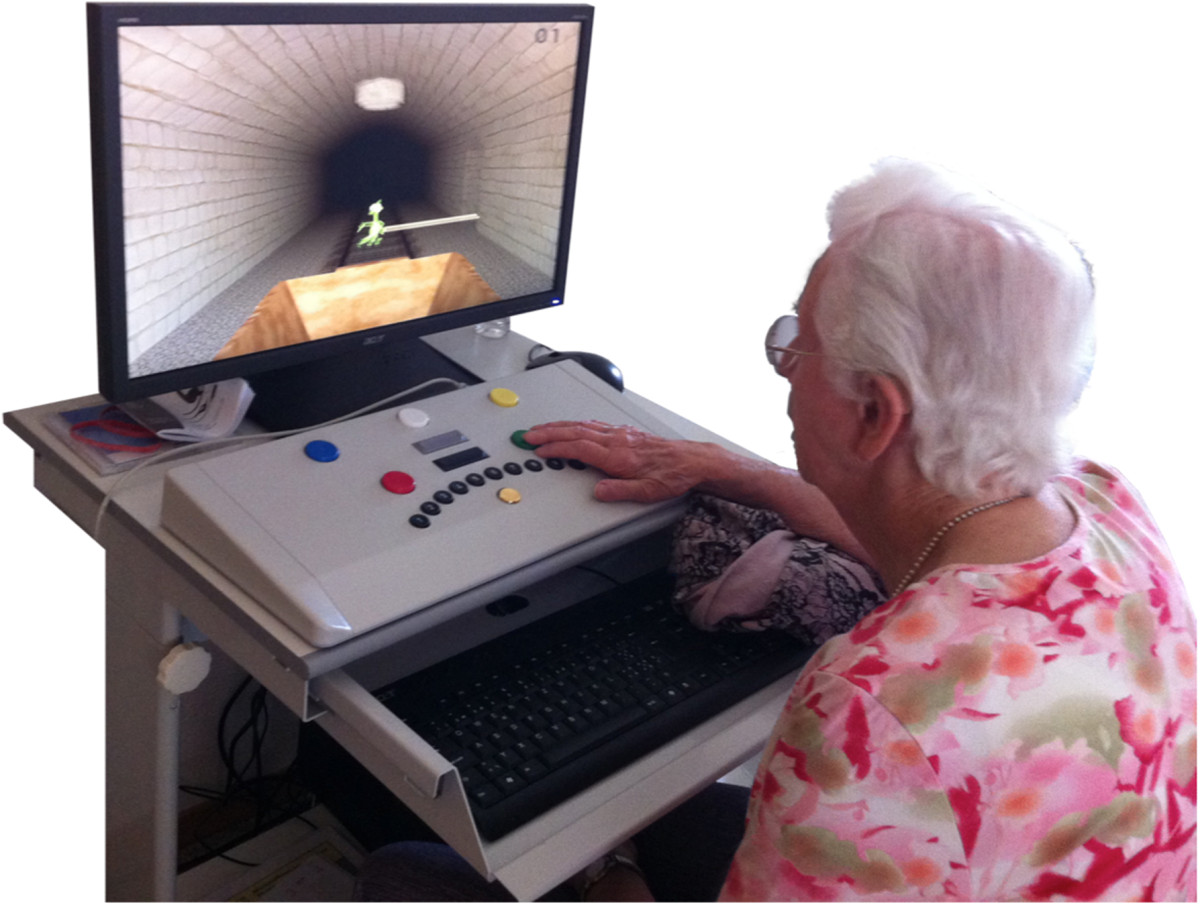
**Exercise example from the cognitive exercise program: a participant training selective attention.**

In addition to the physical training, one group received 12 weeks cognitive training, with the CogniPlus [77] training program (SCHUHFRIED GmbH, 2340 Mödling, Austria, (http://www.schuhfried.at), 3 times a week for 10 minutes. The program was computer-based and supported the training of cognitive abilities (Figure 2). The control group did not have any alternative additional input.

The following tasks for attention training were used: the *Alert* training program trains alertness – the ability to temporarily increase and sustain the intensity of attention; the *Select* training program trains selective attention – the ability to respond quickly to relevant stimuli and to suppress inappropriate responses; the *Divid* training program trains divided attention – the ability to perform different tasks simultaneously.

The ability dimensions were trained using realistic scenarios. In the *Alert* training program, a motorcycle is driven along a road, and the participant’s task was to react as quickly as possible when obstacles appear (e.g. an animal crossing the road), by pressing a reaction key. In the *Select* training program the participants drove through a tunnel in a mine rolley and had to react on relevant visual and/or acoustic stimuli (e.g. yellow birds making a noise pre-defined for that animal) and to suppress reaction on irrelevant stimuli (e.g. a gray mouse making a noise of a bird). During the *Divid* training program the participant’s task was to observe an airport as a security official. The participants had to simultaneously observe different screens with several control monitors (e.g. ticket counter, luggage conveyor) and announcements over the loudspeaker, and to react appropriately on these stimuli.

The training principle of progression was implemented in this part of the training. The intensity of the cognitive training program was progressively increased or decreased, based on the abilities of the performer. When performers adapted to a certain training level, program variables (e.g. speed) were automatically modified. The program has previously shown to be able to improve attention [78].

### Primary outcome

#### Dual task costs of walking

Spatio-temporal walking parameters were measured with the 7.92 meters portable electronic GAITRite® walkway (CIR Systems, Havertown, USA), Platinum Version 4.0 software, a valid and reliable tool for gait analysis in older adults [79]. Subjects were instructed to walk under four different conditions: (1) walk at self-selected speed (*preferred walking*), (2) at fast speed (*fast walking*), (3) at self-selected speed while continuously subtracting sevens or threes from a random given number between 200-250 or while enumerating animals or flowers (*DT preferred walking*), (4) fast walking while continuously subtracting or enumerating (*DT fast walking*). Participants walked two or three trials for each condition. Derived walking parameters were: velocity (m*s-1), step time (s), step length (m) and variability, expressed as standard deviation (SD) of step length (m).

We calculated for each subject and task the relative dual task costs (DTC), as percentage of loss relative to the single-task walking (expressed in absolute values), according 100 * |(single task score – dual task score)/single task score| [80].

### Secondary outcomes

#### Physical performance measure

Physical performance was assessed with the short physical performance battery (SPPB) and the expanded timed get-up-and-go (ETGUG) test. The expanded timed get-up-and-go (ETGUG) test measures times to complete six component tasks identifiable in the TUG test; sit-to-stand, gait initiation, walk 1, turn around, walk 2, slow down, stop, turnaround, and sit down [81]. SPPB is valid and reliable for lower extremity functions [82], and predictive for disability [83]. ETGUG serves as an objective and reliable assessment of functional ability in older adults [84].

#### Simple reaction time

Simple reaction time tasks were used to measure psychomotor speed. Reaction time was assessed using a hand-held electronic timer and a light as the stimulus. Depression of a switch by the finger and the foot served as response [85].

#### Executive functions

The Trail Making Test A & B assesses executive functions, attention, and processing speed, and consists of two parts; TMT-A and TMT-B. TMT-A is a visual-scanning task, and cognitive flexibility is required to conduct TMT-B [86].

#### Divided attention

We assessed divided attention with the computerized Vienna Test System (SCHUHFRIED GmbH, 2340 Mödling, Austria, (http://www.schuhfried.at). The participant receives stimuli on two visual channels. The upper stimulus (upper channel) presented a light grey circle, and the lower stimulus a light grey square (lower channel) on a white screen. The two stimuli appear and disappear continuously, and sometimes one or both of the stimuli change the colour to dark grey. The task was to observe if one of the stimuli has changed from light grey to dark grey two times in series and, in this case, to press the response key [87]. Analysed parameters were: reaction time upper channel (s), reaction time lower channel (s), number of missed answers upper and lower channel.

#### Fear of falling

The Falls Efficacy Scale International (FES-I) was used as a measure of ‘concern’ about falling to determine the transfer effects of training. The FES-I has excellent internal and test-retest reliability [88].

All measurements and the intervention program were conducted in suitable locations at the homes-for-the-aged. Outcome variables were taken at baseline and after 12 weeks of the intervention. Individuals meeting the eligibility criteria took part in a personal questionnaire based interview screening for cognitive and health problems.

#### Falls

Falls, defined as ‘unexpected events in which the participant comes to rest on the ground, floor or lower level’ [89], were assessed from 6 months retrospectively to 12 months prospectively using a fall calendar. Fall rates (falls per month) were analyzed for three periods; 1) 6 months retrospectively to study commencement, 2) 3 months during the study, 3) 12 months following training ending. Retrospective falls were reported at study commencement and based on data information in the data systems of the homes, which was combined with interviews of the trainees. For the other two periods falls calendars were provided to the health care staff of the homes-for-the-aged, filled-in on a weekly basis and returned after a period ended.

### Randomization

To ensure allocation concealment, participants in each home were enrolled by the health care staff, and randomized by the person assessing the outcome measures using simple (unrestricted) randomisation [70] based on a table of random numbers. The assessor generated an unpredictable allocation sequence, which was concealed until assignment occurred. Each participant in every home received a two digit number (01, 02, 03, …) resulting in a rank order of the participants. With the help of the random numbers table the assessor decided a priori to pick a number from the table with a pencil and go through the table either from bottom-right to upper-left in a diagonal way, horizontally from left-to-right or right-to-left, etc. Even and uneven numbers decided group allocation. All individuals were allocated this way to one of the two groups where for each location a different number of the table of random numbers was taken. Because of the sample size achieved we decided not to compare the totals for each group and choose the group that would give most balance overall for the last participants to be included. With this procedure we ended up with a slightly uneven distribution, however, without having to use blocking or stratification. The health care staff assigned participants to the training groups. The intervention was absolved in groups of 4 or 5 and supervised by instructed personnel of the homes-for-the-aged. Blinding of the investigator was not possible because the investigator conducted part of the assessments.

### Statistical analysis

All available data were analyzed by initial group assignment and were performed with an intention to treat approach [90]. All participants (including drop-outs) were integrated in the analysis, regardless of their adherence rate. We assumed that all missing responses were constant and replaced the missing values with mean values of the group to which subjects were originally allocated [91]. A two-way repeated-measure analysis of variance (ANOVA) examined differences between groups and over time. We used pre-post as within-subject factor (2 levels) and groups as between-subject factor (2 levels). A probability level of p < 0.05 was considered significant. A trend to significance was defined as 0.05 < p ≤ 0.10. For effect size, we used η^2^ in ANOVA analyses. Norms for interpreting η^2^ are: 0.01 = small effect, 0.06 = moderate effect and 0.14 = large effect [92]. Sensitivity analyses were performed to deal with outliers [93]. Outliers were excluded using a trimming method [94]. Criteria for outliers based on the interquartile range (IQR), where data below (Q1-1.5*IQR) or above (Q3 + 1.5*IQR) were defined as outliers [95]. All statistical procedures were conducted with SPSS (version 20.00) software (SPSS Inc. Chicago, IL, USA). An attendance rate of 75% and more was deemed acceptable and defined as adherence to the training plan [96].

## Results

**Figure 3:**
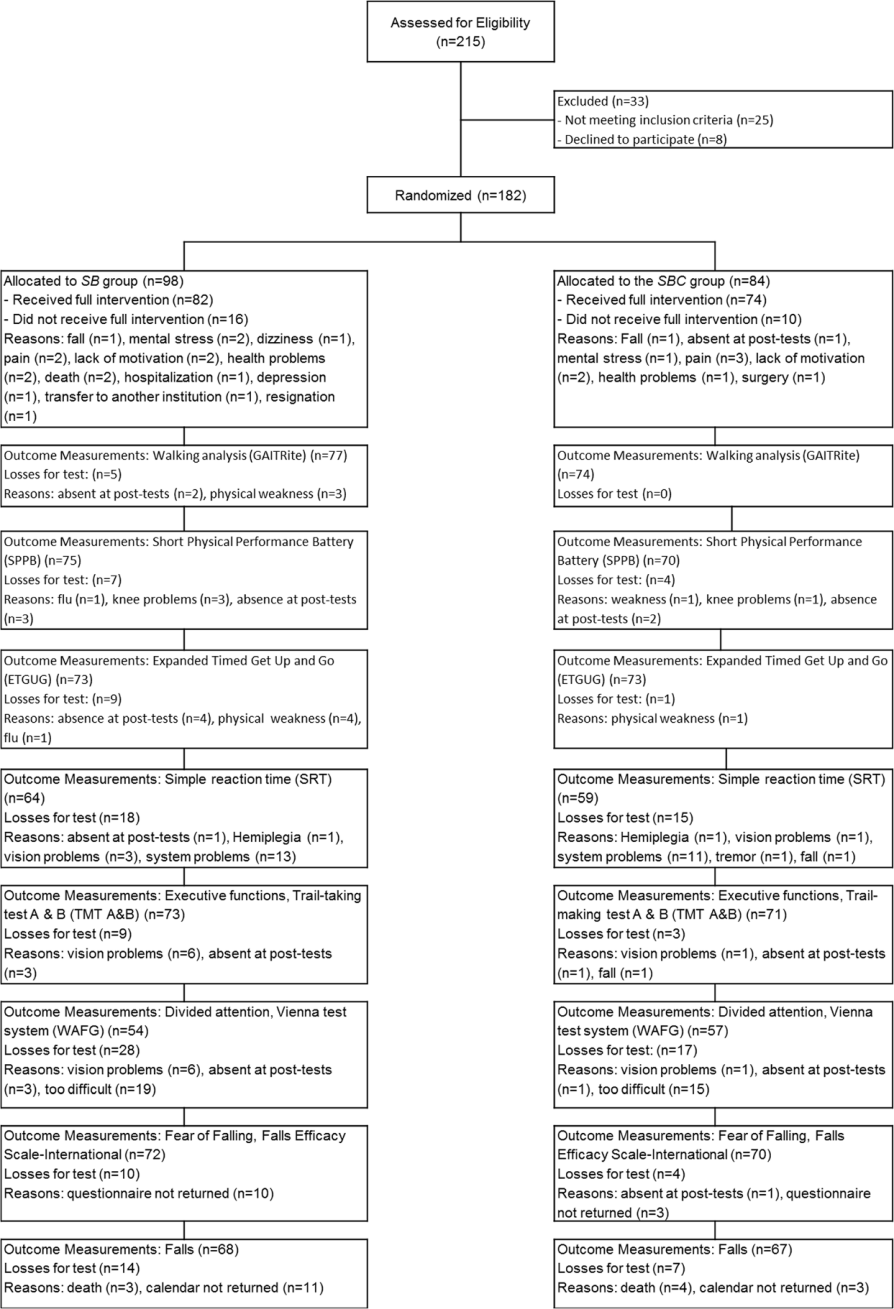
**The study flow chart.**

Variables describing the sample are summarised in Table 1. One hundred eighty two participants fulfilled the initial eligibility criteria and were randomly assigned to either *SB* (94) or *SBC* (88). With the reallocation of 4 participants from *SBC* to *SB* the intervention started with 98 Participants in the *SB* and 84 Participants in the *SBC* group. A total of 156 participants completed the intervention (137 subjects living in the homes-for-the-aged and 19 subjects living in the vicinity) resulting in 14.3% attrition (Figure 3). Adherence to strength-balance training was 91.4% for *SB* (21.9 out of 24 sessions) and 89.5% for *SBC* (21.5 out of 24 sessions). Average adherence to the cognitive intervention was 85.4% (307.4 out of 360 scheduled minutes).

**Table 1:** **Participants’ demographic and baseline characteristics**

Group	***SB***group	***SBC***group
No. of participants with a complete questionnaire	76	69
Age (mean ± SD)	81.9 ± 6.3	81.1 ± 8.3
Sex (female, male)	52, 30	49, 25
MMSE score (mean ± SD)	27.7 ± 2.9	27.6 ± 2.6
**Fall risk factors**		
Slow walking speed (<1.22 m/s) n(%)	64 out of 77(83)	62 out of 74(84)
Fell in the last 6 months n(%)	23(30)	20(29)
3 or more prescription medications n(%)	45(59)	51(73)
Physical functioning; SPPB (mean ± SD)	7.3 ± 2.6	7.3 ± 2.6
Fear of falling; FES-I (mean ± SD)	25.4 ± 8.0	26.8 ± 9.6
**Education/profession n(%)**		
University/College	4(5)	7(10)
Vocational Education	52(68)	41(59)
No educated profession	20(26)	21(30)
In a sitting position past profession	15(20)	18(26)
**Health questions n(%)**		
Number of self-reported chronic diseases		
Joint diseases	35(46)	34(49)
Hypertension	40(53)	37(54)
Cardiac Problems	27(36)	29(42)
Osteoporosis	13(17)	12(17)
Type II diabetes mellitus	9(12)	11(16)
Problems limiting walking function		
Self-reported walking problems	31(41)	34(49)
Problems with legs	40(53)	41(59)
Need walking aid	31(41)	36(52)
Hearing problems	41(54)	35(51)
Vision problems	34(45)	32(46)
Dizziness	28(37)	21(30)
Estimated good health	48(63)	36(52)
Estimated better health compared with contemporary	26(34)	23(33)
Estimated good balance	29(38)	22(32)
Feel pain daily	22(29)	22(32)
**Physical activity questions n(%)**		
Practiced some sport in the past	34(45)	34(49)
Practiced strength exercises in the past	6(8)	5(7)

### Primary outcome

#### Dual task costs of walking

Table 2 demonstrates results of the dual task costs of walking, excluding outliers. The results of sensitivity analyses [93, 97] in addition to the primary intention to treat analyses where outliers are included, and the participants are analysed in the group where they were initially allocated, are reported in an additional file of this manuscript (see Additional file 1).

**Table 2:** **Dual task costs of walking of** ***SB*** **and** ***SBC*** **from pre- to post-test, between-groups differences and interaction effects**

	***SB***group (n = 77)		***SBC***group (n = 74)		pre-post differences (both groups)	between-groups differences	interaction effect
**Conditions** Parameters	Pre-test (mean ± SD)	Post-test (mean ± SD)	Pre-test (mean ± SD)	Post-test (mean ± SD)	p_within_/η2	p_between_/η2	p_interaction_/η2
**DTC preferred**							
Velocity (%)	13.9 ± 17.8	12.2 ± 15.0	17.5 ± 18.4	11.0 ± 17.5	0.002*/0.051	0.588/0.002	0.067°/0.019
Step time (%)	12.0 ± 25.2	9.6 ± 14.4	31.3 ± 88.7	10.0 ± 18.7	0.018*/0.033	0.061°/0.021	0.061°/0.021
Step length (%)	6.6 ± 9.0	6.6 ± 8.4	7.4 ± 9.7	4.4 ± 9.5	0.025*/0.028	0.558/0.002	0.028*/0.028
SD step length (%)	28.7 ± 53.2	27.9 ± 54.2	24.7 ± 50.4	18.0 ± 51.7	0.426/0.004	0.298/0.007	0.531/0.002
**DTC fast**							
Velocity (%)	26.5 ± 13.2	21.9 ± 9.0	28.6 ± 13.3	22.7 ± 13.1	<0.001*/0.126	0.355/0.005	0.545/0.002
Step time (%)	23.1 ± 24.5	17.2 ± 10.1	18.8 ± 12.7	17.5 ± 17.2	0.035*/0.028	0.368/0.005	0.177/0.012
Step length (%)	10.8 ± 8.5	10.3 ± 6.7	13.1 ± 8.5	9.8 ± 8.3	0.001*/0.073	0.426/0.004	0.009*/0.040
SD step length (%)	17.1 ± 34.9	50.3 ± 150.1	25.1 ± 54.7	20.1 ± 43.2	0.311/0.006	0.625/0.001	0.014*/0.036

Notes: * = significant within-groups differences pre-post (p_within_ ≤ 0.05) & significant interactions of the groups (p_interaction_ ≤ 0.05); ° = trends to significant within-groups differences pre-post (0.05 ≥ p_within_ ≤ 0.10), calculated with ANOVA. *Abbreviations:* *DTC* dual task costs, η2: effect size η2 = .01; small effect, η2 = .06; moderate effect, η2 = .14; large effect.

#### DTC preferred speed

Analyses of the DTC at preferred walking speed revealed a significant difference from pre- to post-test for velocity, step time and step length (Table 2). There was a significant interaction for step length (*F*(1,174) = 4.94, *p* = 0.028, η2 = 0.028), in favour of *SBC.*

#### DTC fast speed

The DTC at fast walking speed showed significant differences between pre- and post-test, again for velocity, step time and step length (Table 2). There were significant interactions in favour of *SBC* (step length: F(1,166) = 6.14, *p* = 0.009, η2 = 0.040; SD of step length: F(1,166) = 6.14, *p* = 0.014, η2 = 0.036).

### Secondary outcomes

#### Physical performance measure

The SPPB resulted in a large significant difference over time between pre-test and post-test *F*(1,177) = 227.6, *p* < 0.001, η2 = 0.563: Participants improved their balance, gait initiation, and chair rise performance from pre- (*SB*: 7.33 ± 2.59 points; *SBC*: 7.31 ± 2.61 points) to post-test (*SB*: 9.24 ± 2.30 points, *SBC*: 9.55 ± 1.90 points). There was no significant main effect of group (*p* = 0.661) and no significant interaction effect (*p* = 0.213), suggesting that SPPB performance and the improvements were similar in both groups at all time-points.

The ETGUG total time showed a significant difference over time: pre- and post-test F(1,175) = 77.8, *p* < 0.001, η2 = 0.308, a trend to both a significant effect of group (*p* = 0.052) and an interaction effect (*p* = 0.054). Participants improved their performance from pre-test (SB: 25.86 ± 17.11s; *SBC*: 30.53 ± 17.48s) to post-test (*SB*: 21.10 ± 12.09s; *SBC*: 24.63 ± 11.82). When analysing the component tasks of the test separately, a significant interaction effect F(1,166) = 9.16, *p* = 0.003, η2 = 0.052 emerged for ‘gait initiation’. While *SBC* significantly improved from pre- (2.61 ± 2.18s) to post-test (2.12 ± 1.54s), there was no change for *SB* (pre-test: 1.89 ± 1.23s; post-test: 2.11 ± 2.22s).

#### Simple reaction time

There was a significant effect of training on simple reaction times of both hands and feet (Table 3), with both groups showing decreased RT. Between-groups comparison revealed a significant difference between the groups for the right foot (F(1, 180) = 5.863, *p* = 0.016, η^2^ = 0.032) and no interaction.

**Table 3:** **Pre- and post-test performance for** ***SB*** **and** ***SBC*** **, differences between groups and interaction effects**

	*SB*group		*SBC*group		Pre-post differences (both groups)	Between-groups differences	Interaction effect
	Pre-test (mean ± SD)	Post-test (mean ± SD)	Pre-test (mean ± SD)	Post-test (mean ± SD)	***p***_within_/η^2^	***p***_between_/η^2^	***p***_interaction_/η^2^
**Simple RT**							
RT right hand	362.7 ± 94.8	300.9 ± 57.3	383.0 ± 129.4	318.2 ± 69.0	<0.001*/0.323	0.108/0.014	0.820/0
RT left hand	362.6 ± 88.8	298.4 ± 56.0	374.4 ± 109.6	318.2 ± 74.6	<0.001*/0.339	0.145/0.012	0.524/0.002
RT right foot	423.5 ± 119.3	345.9 ± 67.2	472.5 ± 218.1	380.9 ± 101.1	<0.001*/0.273	0.016*/0.032	0.500/0.003
RT left foot	410.1 ± 110.0	354.3 ± 83.4	442.2 ± 158.7	370.0 ± 82.8	<0.001*/0.261	0.105/0.015	0.312/0.006
**Fear of falling**							
FES-I	25.4 ± 8.0	22.8 ± 7.0	26.8 ± 9.6	24.6 ± 8.5	<0.001*/0.159	0.157/0.011	0.637/0.001
**Executive functions**							
TMT A	83.4 ± 51.4	71.2 ± 47.3	81.5 ± 51.3	69.5 ± 43.4	<0.001*/0.143	0.795/0	0.969/0
TMT B	188.5 ± 73.0	166.3 ± 75.0	189.5 ± 78.8	164.4 ± 76.9	<0.001*/0.207	0.964/0	0.772/0.001
**Divided attention**							
RT upper channel	940.1 ± 170.0	885.7 ± 169.9	1014.0 ± 209.7	907.4 ± 211.3	<0.001*/0.217	0.066°/0.019	0.023*/0.028
RT lower channel	976.7 ± 181.5	893.1 ± 169.2	1012.3 ± 25.0	889.8 ± 204.9	<0.001*/0.292	0.542/0.002	0.105/0.015
MA upper channel	10.7 ± 4.6	10.1 ± 5.0	13.3 ± 5.9	10.0 ± 5.5	<0.001*/0.082	0.064°/0.019	0.001*/0.062
MA lower channel	13.5 ± 5.8	11.5 ± 5.7	15.0 ± 7.2	11.4 ± 6.2	<0.001*/0.161	0.379/0.004	0.094°/0.015

Notes: *significant within-groups differences pre-post (*p*_within_ ≤ 0.05), significant between-groups differences (*p*_between_ ≤ 0.05) & significant interactions of the groups (*p*_interaction_ ≤ 0.05); °trends to significant within-groups differences pre-post (0.05 ≥ *p*_within_ ≤ 0.10) & trends to significant interactions of the groups (0.05 ≥ *p*_interaction_ ≤ 0.10);, calculated with ANOVA. *Abbreviations:* *η2* effect size η2 = .01, small effect, η2 = .06; moderate effect, η2 = .14; large effect, *RT* reaction time, *FES-I* Falls Efficacy Scale-International, *MA* missed answers.

#### Executive functions

Improvements over time of both parts of the trail making test (A and B) were significantly affected by training (Table 3). There was no difference between *SB* and *SBC* and no interaction for this parameter.

#### Divided attention

The reaction times of the test program for divided attention were separately reported for the upper and the lower stimuli channel. There was a significant training related improvement over time in reaction time of both the upper and the lower channel (Table 3), and a significant interaction for the upper channel (F(1,180) = 5.243, *p* = 0.023, η^2^ = 0.028), in favour of *SBC*. Analysis of the number of missed answers revealed significant improvements over time for the groups together and a significant interaction for the upper channel (F(1,180) = 11.839, *p* = 0.001, η^2^ = 0.062), in favour of *SBC*.

#### Fear of falling

There was a significant effect of training from pre- to post-test for FES-I (Table 3) for the whole group. No differences were observed between *SB* and *SBC* and there was no interaction.

#### Falls

**Figure 4:**
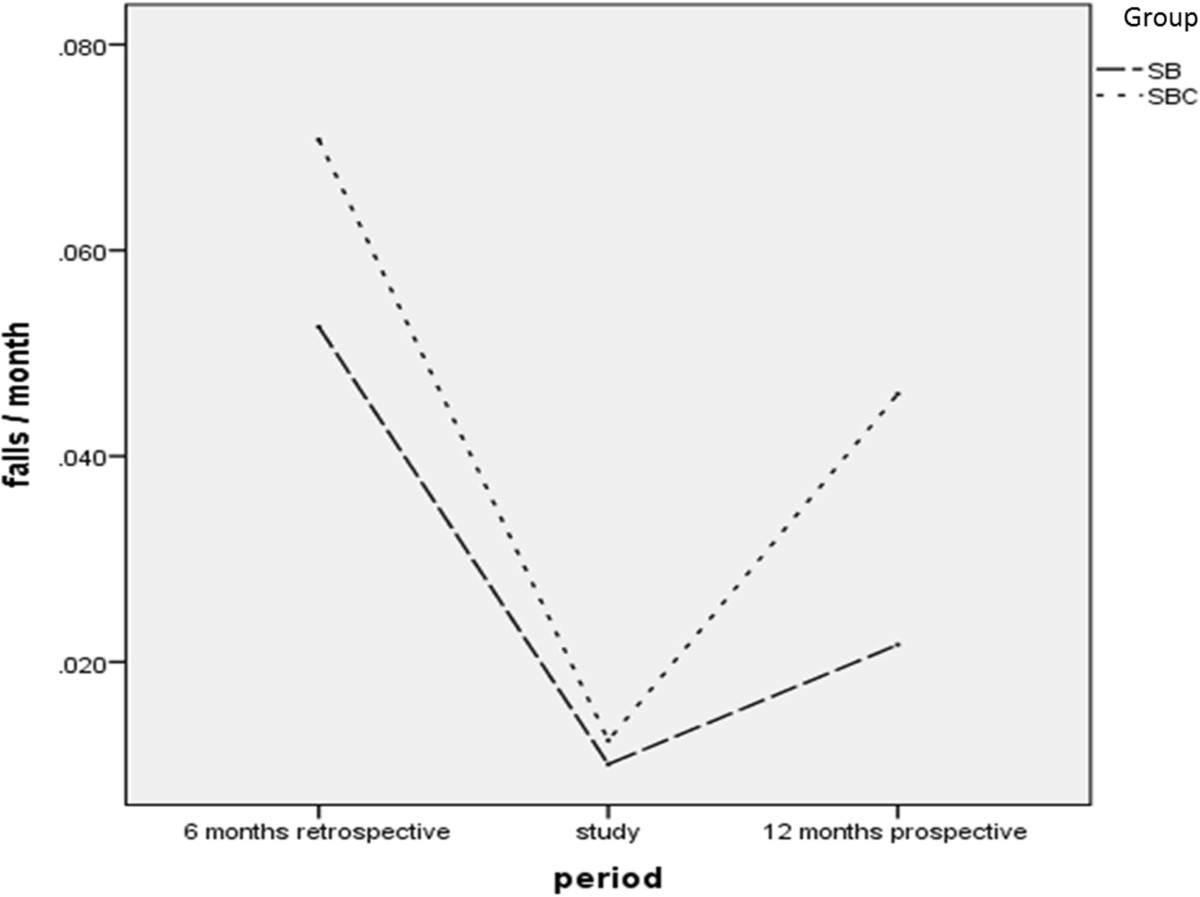
**Falls per month for** ***SB*** **and** ***SBC*** **.** Legend: *SB* and *SBC* prior to study commencement (1; 6 months), during the study (2; 3 months), and after study completion (3; 12 months).

An average of 0.052 ± 0.08 falls per month for *SB* and 0.071 ± 0.1 falls per month for *SBC* were retrospectively (6 months) observable. In the intervention period 0.01 ± 0.047 falls for *SB* and 0.012 ± 0.073 for *SBC* occurred (3 months), and within 12 months following the intervention 0.022 ± 0.040 falls for *SB* and 0.046 ± 0.070 for *SBC* occurred (Figure 4). Fall rate was reduced by 81% for *SB* and 83% for *SBC* during the intervention training period, and by 58% and 46% for *SB* and *SBC* respectively at 12 months follow-up. Effect of time was highly significant from retrospective-to-training F(1,177) = 44.73, *p* < 0.001, η^2^ = 0.202, from retrospectively-to-prospectively F(1,177) = 16.844, *p* < 0.001, η^2^ = 0.087, and over the whole time frame retrospectively-training-prospectively F(1,177) = 28.733, *p* < 0.001, η^2^ = 0.140. There was no significant interaction between the groups for the falls parameter, however, there were significant between groups differences (retrospectively-to-prospectively: F(1,177) = 5.569, *p* = 0.019, η^2^ = 0.031; retrospectively-training-prospectively: F(1,177) = 4.202, *p* = 0.042, η^2^ = 0.023).

## Discussion

This randomized controlled trial examined whether a twelve-week strength-balance exercise regimen, supplemented with computerised cognitive training, would lead to greater improvements in dual task costs of gait, in physical and in cognitive performance compared to strength-balance exercise alone. The study also aimed at exploring the effect on fear of falling and fall rate. We expected improvements in measures of dual task gait, executive functions, and in particular – divided attention, mainly in the strength-balance-cognitive group. In addition, we hypothesized observing different levels in falls behaviour between the groups. Although both groups attained improvements in physical and cognitive performance, the results suggest positive interaction effects for dual task costs of walking and divided attention, in favour of *SBC*. The findings support the notion that it is advantageous to combine physical and cognitive training into clinical practice. The combination seems to have a positive influence on older adults walking abilities under dual task conditions compared to more traditional exercise [67].

Findings from a systematic review demonstrate that a strength and balance exercise regimen is able to preserve or enhance walking abilities [62]. The goal of this study, however, was to optimize walking under dual task conditions as expressed through minimized DTC of walking. Previous findings suggest that resistance training alone has the potential to improve cognitive functions, and particularly executive functions [30, 98]. However, the results of studies with similar groups performing similar strength-balance training, revealed no changes in DTC of walking [99, 100]. When training in combination with video games such improvements are believed achievable [101]. We demonstrated in this study an additional effect of our cognitive program in the sense that the DTC of walking were minimized especially in the *SBC* group. The significant interaction effects observed for step length and step length variability favouring *SBC* extends previous work providing evidence for an association between DTC of step length during fast walking and divided attention [60] into a causal relation. That the group training cognitive skills improved on this measure is reasonable since changes in brain structure associate with reduced gait speed that partly results from shorter steps [102]. Interventions focussing on brain health seem, therefore, important when the aim is to improve gait [102]. The assumption that older adults that fall show shorter step lengths and higher variability compared to non-fallers [103] strengthens the importance of the improvements in these walking parameters of *SBC*. The results of our study are in line with reviews and intervention studies supporting the combination of cognitive and motor programs to attain beneficial effects on DTC of walking compared to more traditional interventions [46, 67, 101]. With a thrice-weekly ten-minute cognitive training focussing on alertness, selective and divided attention, combined with strength-balance exercises, DTC of walking can be minimized.

We found a significant improvement in SPPB scores within both groups reflecting enhanced lower extremity function and walking ability [104]. On average, a person that reaches less than 10 points on the SPPB is almost 3.5 times more susceptible to suffer from mobility disability than a person scoring the maximum of 12 points [104]. At the beginning of training both the *SB* and *SBC* groups reached a mean score of less than 7.5 points, however, they both increased towards 9.3 resp. 9.5 points. Improved gait initiation was only observed for *SBC*. The fact that this intervention impacted on gait initiation is important. Gait initiation is frequently repeated during daily activities, leading to accidental falls during the step initiation phase in people with deficits in balance control [105] and relates to the quality of fronto-striatal brain connections [106]. Stable and efficient mechanisms of the central nervous system (CNS) are required for the control of posture during gait initiation. These mechanisms are complex and require efficient peripheral sensory detection and afferent nerve conduction, followed by central neural processing and efferent nerve conduction [107]. Within older adults, there seems to be a loss of efficiency in these mechanisms leading to falls during gait initiation [108]. It can be hypothesized that by the use of the computerised cognitive training acquired skills led to transfer effects in gait initiation.

The link between cognitive functioning, gait, and the potential for falls was previously established [3]. Specifically, poor EF and attention control, one of the core EFs [109], seemed to be related to fall risk and mortality [51, 52, 110]. Although both our groups were able to improve cognitive functioning as expressed through improvements in reaction time as well as EF, only the group receiving the additional computerized cognitive intervention improved in divided attention skills. Thus, in line with other authors, we demonstrated that falls prevention programs have a positive impact on EF [111], however, the findings also support the assumption that specificity of training applies to these specific EFs. EFs are trainable by repeated practice and with a progressive exercise intensity design at any age [109]. Where the physical training group improved more global measures of cognitive functioning, only the combined training group exhibited training specific improvements.

The non-significant interactions for several cognitive and physical parameters between the groups indicate that both groups improved equally. This seems reasonable because previous research literature describing promotion of resistance training indicated improved cognitive functioning, enhanced functional brain plasticity [30], and altered trajectory of cognitive decline in older adults with probable cognitive deteriorations [112]. Increased performance in selective attention and executive cognitive function for example – achieved through resistance training - has been related to higher walking velocity [98], which in turn relates to improved EFs [113]. Reductions in walking velocity, in general, correlate with declined cognitive factors (e.g. attention and psychomotor speed), falls, and mortality [114–117].

The clinical relevance of improved divided attention might be influencing falls rate in elderly because this function was previously shown to be related to gait and to falls [60]. Our findings, however, reveal no additional effect of training this specific cognitive aspect when it comes to falls. Both training groups improved on the falls parameter with similar magnitudes. Fall rate was reduced in both groups by more than 80% during the intervention period, and by more than 40% during the following 12 months. These results are similar or superior to other interventions incorporating strength and balance exercises [118] and present a clinically relevant reduction in fall risk. Furthermore, our findings confirm the findings of a systematic review including 54 randomised controlled trials showing that exercise programs that combine strength and balance training of sufficient quality can reduce falls with 38% [119]. Our findings compare favourable to other studies that added training components in the sense that the addition of a cognitive component did not lead to a lower effect on falls rate [119]. Unsurprisingly, the lowest fall rate was observed during the study, when compliance was warranted, considering the link between executive functions, gait and falls, and the assumption that poor treatment adherence is related to poor EFs [109]. The fall rate was higher after study termination, however, still significantly lower than prior to study commencement.

Although not more effective in terms of fall events observed, applying a combination of cognitive-motor training might be advantageous to move safer in challenging environments [46, 120] and, therefore, reduces fall risk. We assume this given the additional positive effect of the cognitive intervention on divided attention. To react adequately under circumstances where attention is divided is an important requirement in most activities of daily life. Therefore, with the focus on physical and cognitive improvements in complex situations and the execution of attention-demanding tasks, strength-balance training should be combined with cognitive training.

The results of the sensitivity analysis for DTC of preferred walking were not robust to the exclusion of outliers and changed when they were excluded. The primary analysis, shown in the Additional file 1, revealed a statistical interaction effect for DTC of velocity favouring *SBC*, which was not significant in the analysis where outliers were excluded. The non-significant interaction for SD of step length in our analysis with the outliers included demonstrated a trend to statistical significance in the primary analysis. The differences in mean values and standard deviations of the groups observable between the analyses implies that the results of the primary analysis were affected by the outliers [93]. Removing these participants from the analysis was legitimate to avoid bias and to minimize random error [94, 121].

In this trial, the dual task costs of walking were assessed. Not, however, the cognitive dual task costs while walking. To assess the possible effects of our program on cognitive functions we resorted to specific cognitive tests. In our trial the main interest was the effect of an attention-demanding task on gait performance. Participants were instructed not to prioritize one task (walking) over the other (calculating) but to try and perform both as good as possible at the same time. The ability of counting backwards was not used as an outcome measure to determine the effect of training on cognitive performance, thus, causing the reliability of this instruction for reproduction purposes being of lesser importance for our study. The only reason for using the counting task was to disturb the gait pattern of our subjects and, by doing that, determine the dual task costs of walking. Allowing both gait and cognitive task performance to vary has previously been shown to better represent the dynamics of daily living tasks of older adults [122, 123] and is, furthermore, a reliable procedure to determine dual task costs of walking even in older adults with mild cognitive impairments [124].

An obvious strength of our study is the rather large sample size minimising the chance of type I and II errors. This study, therefore, reveals credible estimates for these measures because it is sufficiently powered. However, when evaluating the validity of a study it is important to consider both the clinical and statistical significance of the parameters [125]. Researchers and clinicians should not focus solely on small P-values to decide whether a treatment is clinically useful, but should also consider the magnitudes of treatment differences [125]. The majority of the between groups comparisons for fast walking show small-to-moderate magnitudes of treatment differences and should, accordingly, lead to a cautionary interpretation. The relationship between physical and cognitive training research and its effect on gait in older adults requires further exploration. A possible explanation for these small-to-moderate effect sizes might be caused by the implementation of cognitive training. The advantages of computerized training programs are documented in recent work [67, 126]. In our program the motor and the cognitive part were offered as separate entities consecutively. There is increasing evidence, however, that simultaneously performed cognitive-motor programs are more effective in influencing both cognitive and motor functioning [34, 127]. The individual and combined effects of physical and mental exercise interventions reported cognitive benefits to be larger with the combined cognitive and physical training paradigms [128, 129].

### Limitations

This study has several limitations. As already discussed the small-to-moderate effect sizes should be considered when interpreting data. The small magnitudes of the interaction effects give rise to possible bias in our research design [130]. We treated the dropouts of this study as a part of the treatment group to which they were assigned even if they did not receive the full intervention. Intention to treat is a recommended approach to several types of non-adherence to the study protocol [131], able to reduce the potential drop out bias effect [132]. We replaced missing data with the mean values of the groups, thus allowing complete case analysis. A drawback of this approach is reduced variability and weakening of covariance and correlation estimates in the data. We excluded outliers with a trimming method, which is a method applied when good reasons to believe that the subject(s) with the extreme value(s) was/were not from the same population [94] exist. The intention to treat analysis was not robust for some values of gait analysis with the outliers included. In particular the results for the SD for variability data (expressed as SD of step length) were different between the sensitivity analyses. A potential reason for outliers in the datasets is that the participants differed in baseline characteristics. One of our inclusion criteria was “able to walk 20 meters with or without walking aid”, thus, all people able to walk were included, independent of their walking characteristics (e.g. walking velocity or instability).

To move 4 participants from *SBC* to *SB* was based on a similar consideration in order to avoid a random error, and has, potentially, the same origin [121]: We only registered “vision problems” in the baseline demographics of the participants. The ability to follow a game on a computer screen was not mentioned as inclusion criteria, which might be considered for future studies.

Furthermore, the study contained the training of three different dimensions of attention as cognitive training. It warrants further research to examine which program/s was/were the reason for the examined results. An obvious limitation was that the test for divided attention was too difficult for several participants, leading to floor effects and multiple losses for the test. The interaction effect for measures of divided attention should also be interpreted cautiously, since magnitude of treatment differences is small-to-moderate.

## Conclusions

Both strength-balance and strength-balance-cognitive training enhanced physical performance, reaction time, executive functions, and reduced fall rate and fear of falling substantially. Only strength-balance-cognitive training reduced dual task costs of walking and improved gait initiation, and divided attention was merely improved by the cognitive-motor group. The larger improvements in divided attention and dual task walking highlight that an exercise program aiming at improving tasks that require attentional control should include a cognitive challenging element. This study may constitute a reference for further studies in the topic of fall prevention in older adults with the aim to improve physical performance under dual task conditions, and to reduce falls. Future studies are advised to compare different types and modes of exercise where different specific perceptual and cognitive demands are to be considered in the research design; e.g. complementary motor and cognitive training paradigms against integrative motor-cognitive training approaches.

## Electronic supplementary material

Additional file 1: **Sensitivity analysis for dual task costs of walking.** Dual task costs of walking of *SB* and *SBC* from pre- to post-test, between-groups differences and interaction effects for the intention to treat analysis. All outliers are included. The participants that were reallocated from the *SBC* to the *SB* group (due to vision problems) are analysed as participants from *SBC* group (as initially allocated). (DOCX 16 KB)

Below are the links to the authors’ original submitted files for images.

Authors’ original file for figure 1

Authors’ original file for figure 2

Authors’ original file for figure 3

Authors’ original file for figure 4
