# Supplementary material for: Strength-balance supplemented with computerized cognitive training to improve dual task gait and divided attention in older adults: a multicenter randomized-controlled trial
Source: BMC Geriatr. 2014 Dec 15;14:134. doi: 10.1186/1471-2318-14-134 (PMC4293005; doi:10.1186/1471-2318-14-134)
Supplement: Supplementary file 1 — Additional file 1: Sensitivity analysis for dual task costs of walking. Dual task costs of walking of SB and SBC from pre- to post-test, between-groups differences and interaction effects for the intention to treat analysis. All outliers are included. The participants that were reallocated from the SBC to the SB group (due to vision problems) are analysed as participants from SBC group (as initially allocated). (DOCX 16 KB) [file 12877_2014_1077_MOESM1_ESM.docx]

|  | ***SB* group** | |  | ***SBC* group** | |  | **pre-post differences (both groups)** |  | **between-groups differences** |  | **interaction effect** |
| --- | --- | --- | --- | --- | --- | --- | --- | --- | --- | --- | --- |
| **Conditions** Parameters | Pre-test (mean±SD) | Post-test (mean±SD) |  | Pre-test (mean±SD) | Post-test (mean±SD) |  | p_within_/η2 |  | p_between_/η2 |  | p_interaction_/η2 |
| **DTC preferred** |  |  |  |  |  |  |  |  |  |  |  |
| Velocity (%) | 13.0±16.8 | 12.1±15.1 |  | 18.3±19.2 | 11.2±17.3 |  | 0.002* / 0.051 |  | 0.330 / 0.005 |  | 0.019* / 0.031 |
| Step time (%) | 13.6±33.4 | 11.2±26.6 |  | 33.2±88.2 | 11.9±23.2 |  | 0.014* / 0.034 |  | 0.083° / 0.017 |  | 0.053° / 0.021 |
| Step length (%) | 5.8±9.7 | 5.9±8.8 |  | 7.8±10.0 | 4.8±9.9 |  | 0.033* / 0.026 |  | 0.733 / 0.001 |  | 0.021* / 0.030 |
| SD step length (%) | 30.5±57.8 | 28.5±54.7 |  | 69.4±225.1 | 24.0±60.0 |  | 0.058° / 0.021 |  | 0.206 / 0.009 |  | 0.082° / 0.017 |
| **DTC fast** |  |  |  |  |  |  |  |  |  |  |  |
| Velocity (%) | 25.7±12.1 | 21.9±9.1 |  | 29.3±14.1 | 22.7±12.9 |  | <0.001* / 0.128 |  | 0.157 / 0.012 |  | 0.18 / 0.011 |
| Step time (%) | 21.8±27.3 | 16.8±12.8 |  | 28.7±31.7 | 21.7±24.4 |  | 0.003* / 0.052 |  | 0.077° / 0.019 |  | 0.606 / 0.002 |
| Step length (%) | 10.7±8.5 | 10.3±6.7 |  | 13.4±8.7 | 10.2±8.9 |  | 0.001* / 0.068 |  | 0.263 / 0.008 |  | 0.008* / 0.042 |
| SD step length (%) | 16.5±35.3 | 51.9±149.5 |  | 39.3±107.7 | 21.2±42.7 |  | 0.38 / 0.005 |  | 0.723 / 0.001 |  | 0.007* / 0.043 |
| Notes: * = significant within-groups differences pre-post (p_within_ ≤ 0.05) & significant interactions of the groups (p_interaction_ ≤ 0.05); ° = trends to significant within-groups differences pre-post (0.05 ≥ p_within_ ≤ 0.10), calculated with ANOVA. Abbreviations: DTC; dual task costs, η2: effect size η2=.01; small effect, η2=.06; moderate effect, η2=.14; large effect | | | | | | | | | | | |
