# Supplementary figures and images for: Strength-balance supplemented with computerized cognitive training to improve dual task gait and divided attention in older adults: a multicenter randomized-controlled trial
Source: BMC Geriatr. 2014 Dec 15;14:134. doi: 10.1186/1471-2318-14-134 (PMC4293005; doi:10.1186/1471-2318-14-134)

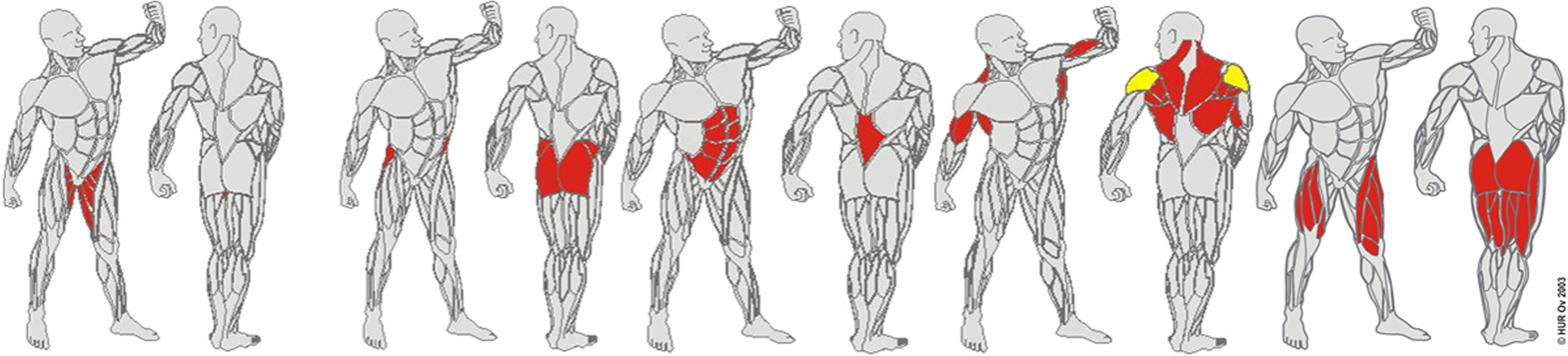

Supplement: Supplementary file 2 — Authors’ original file for figure 1 [file 12877_2014_1077_MOESM2_ESM.tif]

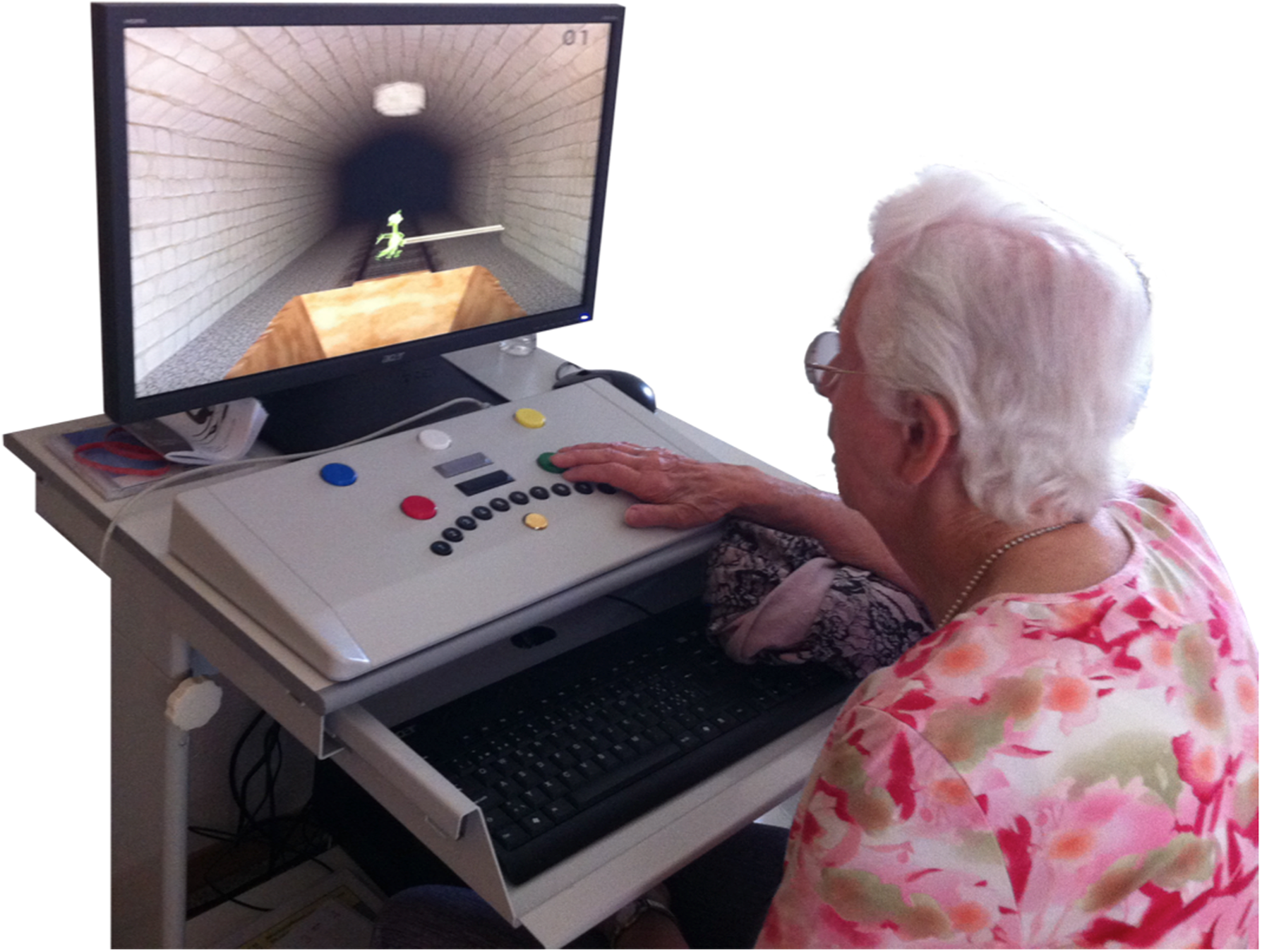

Supplement: Supplementary file 3 — Authors’ original file for figure 2 [file 12877_2014_1077_MOESM3_ESM.tif]

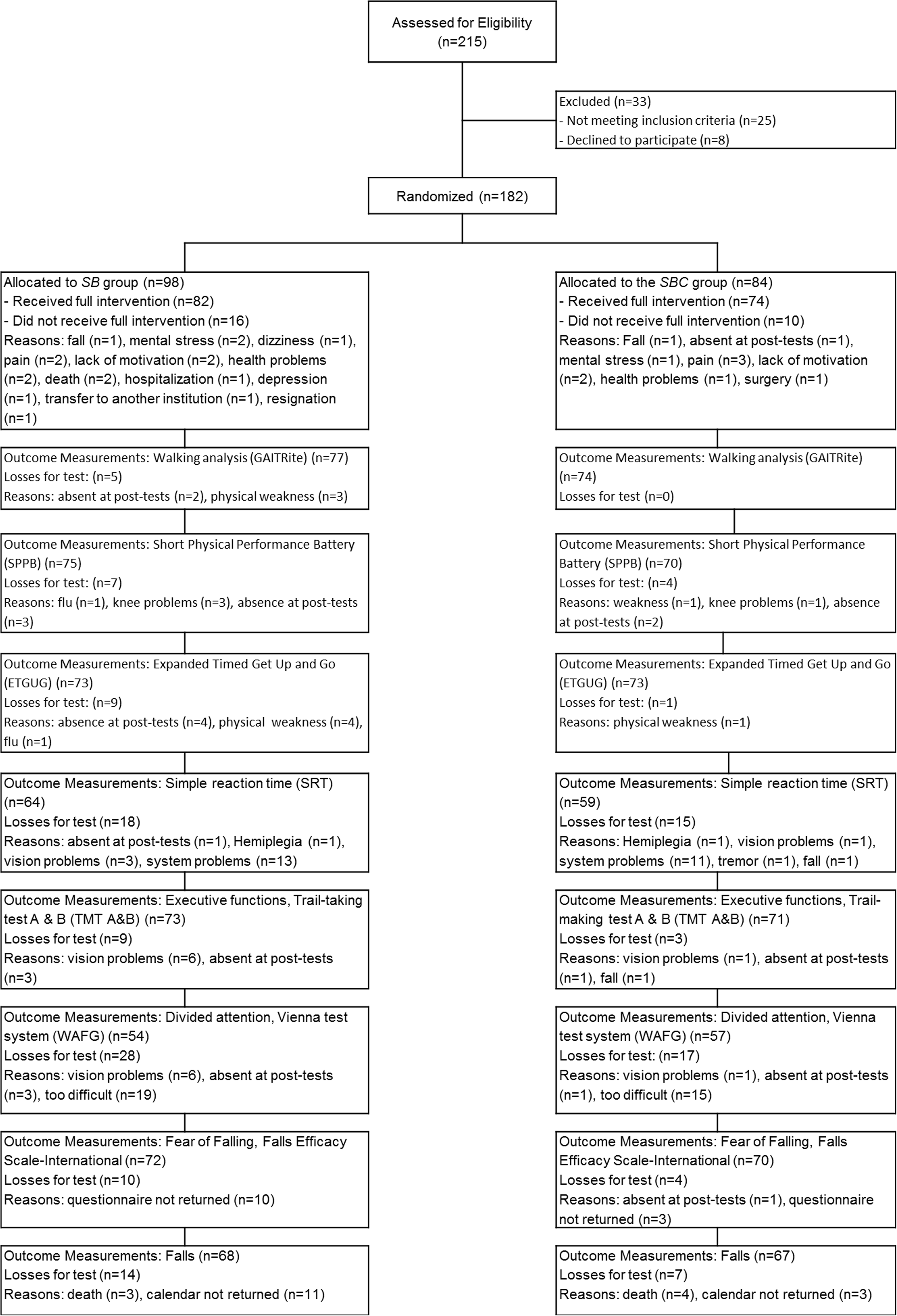

Supplement: Supplementary file 4 — Authors’ original file for figure 3 [file 12877_2014_1077_MOESM4_ESM.tif]

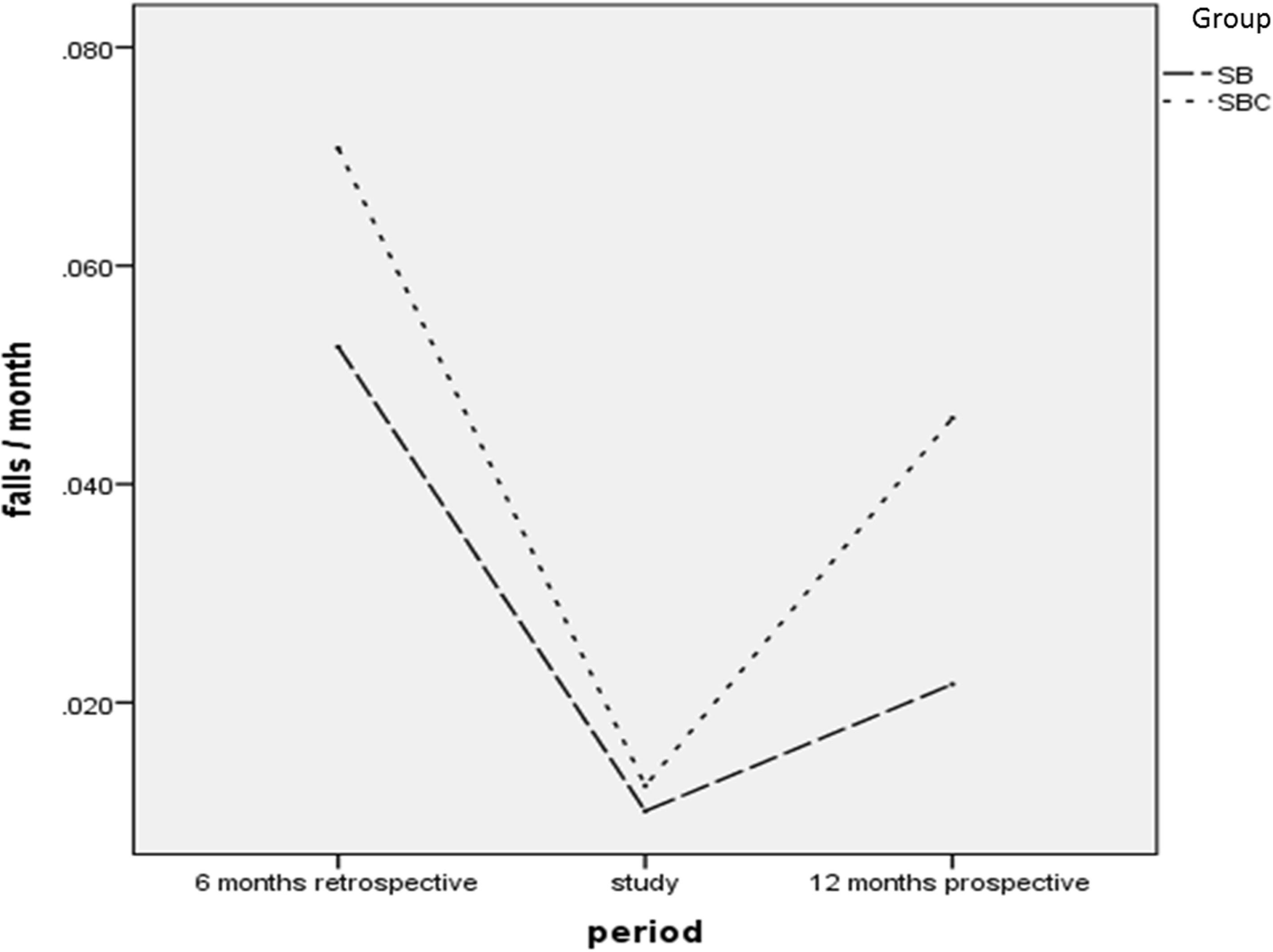

Supplement: Supplementary file 5 — Authors’ original file for figure 4 [file 12877_2014_1077_MOESM5_ESM.tif]
